# Transcriptomic Insights into the Association of IL-1 Signaling with the Senescence-Associated Secretory Phenotype in Human Fibroblasts

**DOI:** 10.3390/genes17050527

**Published:** 2026-04-29

**Authors:** Vural Yilmaz

**Affiliations:** Biotechnology Research Center (BRC), Cyprus International University (CIU), Via Mersin 10, Nicosia 99258, Northern Cyprus, Türkiye; vyilmaz@ciu.edu.tr

**Keywords:** cellular senescence, senescence-associated secretory phenotype, interleukin-1 signaling, replicative senescence, transcriptomic network analysis

## Abstract

Background/Objectives: Cellular senescence is a stable growth-arrested state accompanied by the senescence-associated secretory phenotype (SASP), a complex inflammatory secretome that contributes to tissue remodeling, chronic inflammation, and age-related disease. Although multiple signaling pathways have been implicated in SASP regulation, the extent to which interleukin-1 (IL-1) signaling is associated with the organization of SASP-associated transcriptional programs remains incompletely defined at the transcriptomic level. Methods: Here, we performed a focused in silico analysis of a publicly available RNA-sequencing dataset (GSE63577) profiling primary human fibroblasts undergoing replicative senescence. Differential expression analysis revealed broad inflammatory remodeling in senescent fibroblasts, including robust upregulation of canonical SASP-associated cytokines, chemokines, and matrix-related factors. Targeted visualization using a curated, literature-defined SASP gene panel confirmed consistent transcriptional activation of key SASP components during replicative senescence. Results: To assess transcriptional associations, we performed correlation-based network analysis centered on *IL1A* and *IL1B*. This analysis demonstrated strong transcriptional coupling between IL-1 signaling components, NF-κB-related genes, and SASP-associated transcripts, revealing a highly connected inflammatory module embedded within the senescence transcriptome. Pathway-level integration using curated gene sets further highlighted IL-1 signaling, cytokine signaling, and NF-κB-related pathways as dominant features of senescence-associated transcriptional changes. These patterns were further supported by analysis of an independent fibroblast senescence dataset (GSE41714), demonstrating consistent IL-1-associated and SASP-related transcriptional trends across experimental systems. Conclusions: Together, these findings suggest that IL-1 signaling is consistently associated with a central position within the SASP-associated transcriptional network during replicative senescence in human fibroblasts. Therefore, the present study contributes transcriptomic network-level evidence supporting an association between IL-1 signaling and coordinated SASP-associated inflammatory programs, and highlights its potential relevance for intervention strategies.

## 1. Introduction

Cellular senescence is a stress-response program in which cells enter a durable proliferative arrest accompanied by extensive remodeling of chromatin organization, metabolism, and intracellular signaling. While senescence plays beneficial roles in tumor suppression, tissue repair, and embryonic development, the progressive accumulation of senescent cells with age has been strongly implicated in chronic inflammation, tissue dysfunction, and a wide range of age-related pathologies. Central to these detrimental effects is the senescence-associated secretory phenotype (SASP), a complex and dynamic secretory program composed of pro-inflammatory cytokines, chemokines, growth factors, extracellular matrix-remodeling enzymes, and other bioactive molecules that can profoundly influence the surrounding tissue microenvironment and systemic physiology [1,2,3,4].

The SASP is now recognized as the principal mediator of the non-cell-autonomous effects of senescence, driving immune cell recruitment, paracrine induction of senescence, and persistent inflammatory signaling that contributes to “inflammaging” and disease progression [1,2,3]. Importantly, the SASP is not a uniform entity. Its composition varies depending on cell type, senescence-inducing stimulus, duration of senescence, and tissue context, resulting in substantial heterogeneity across experimental systems [1,5,6,7,8]. Large-scale proteomic and transcriptomic profiling efforts have demonstrated that only a subset of inflammatory mediators is consistently elevated across senescent states, whereas many SASP components are context-dependent [5]. This heterogeneity has important implications for both biomarker discovery and therapeutic strategies aimed at modulating senescence-associated inflammation without eliminating senescent cells entirely [1,2,3]. Among SASP-producing cell types, fibroblasts represent a particularly influential source due to their abundance, longevity, and prominent role in shaping tissue inflammatory microenvironments.

Among the regulatory pathways implicated in SASP control, interleukin-1 (IL-1) family signaling and nuclear factor kappa B (NF-κB)-dependent transcriptional programs have repeatedly emerged as central inflammatory axes. Seminal studies in human fibroblasts demonstrated that cell surface-associated IL-1A acts as an upstream regulator of the IL-6/IL-8 cytokine network, functioning through sustained NF-κB activation and establishing a self-reinforcing inflammatory loop [9,10,11]. Additional work has shown that inflammasome-dependent IL-1 signaling contributes to the orchestration of broader secretory programs and promotes paracrine senescence, further supporting a key role for IL-1-mediated pathways in senescence-associated inflammation [12]. At the same time, regulatory mechanisms such as mTOR-dependent control of IL-1α translation indicate that IL-1 signaling output can be modulated at multiple molecular levels during senescence [13]. Despite these advances, it remains unclear how IL-1 signaling is positioned within the broader transcriptional architecture of the SASP, and whether it exhibits features consistent with a central hub-like role at the transcriptomic level.

The relevance of IL-1 signaling in senescence is further underscored by its broader involvement in chronic inflammatory diseases and cancer. Persistent IL-1B signaling has been implicated in shaping pro-tumorigenic microenvironments through effects on angiogenesis, immune cell recruitment, and extracellular matrix remodeling [14]. Although IL-1A and IL-1B may play distinct roles in senescent cells, particularly in fibroblast systems where IL-1α is often described as a proximal SASP trigger, both cytokines converge on shared receptor-proximal signaling components and NF-κB activation, supporting pathway-level analyses of IL-1 signaling in the context of senescence [9,10,12,13,14].

Primary human fibroblasts constitute one of the most extensively characterized experimental systems for studying cellular senescence and SASP regulation. These cells undergo replicative senescence following repeated population doublings, accompanied by reproducible transcriptional and phenotypic changes that have been documented across multiple independent studies [15,16,17]. Among publicly available transcriptomic resources, the RNA-sequencing dataset GSE63577 provides a well-defined comparison of early-passage and senescent human fibroblasts generated under the JenAge consortium framework [15,18]. This dataset has been widely used to identify conserved senescence-associated genes and pathways, making it a robust platform for focused, hypothesis-driven analyses of senescence-associated transcriptional programs [15].

While genome-wide analyses have revealed extensive transcriptional remodeling during senescence, there remains value in targeted, mechanistically motivated re-analyses that address specific biological questions with high interpretability. In particular, a systems-level understanding of how IL-1-associated transcriptional programs are embedded within the broader SASP network, rather than solely their individual regulatory roles, remains incompletely explored. In this context, and building upon prior work, including our own analyses of IL-1B signaling in inflammatory and disease settings [14], we sought to examine the transcriptomic relationship between IL-1 signaling components and a curated SASP gene program in human fibroblast senescence.

Here, we test the hypothesis that IL-1 signaling is associated with a central, hub-like position within the senescence-associated secretory phenotype at the transcriptomic level in human fibroblasts.

## 2. Materials and Methods

### 2.1. Transcriptomic Dataset and Study Design

Publicly available transcriptomic data were obtained from the Gene Expression Omnibus (GEO) under accession number GSE63577, which contains RNA sequencing-based expression profiles of human fibroblasts undergoing replicative senescence. In this study, analyses were restricted to human foreskin fibroblasts (HFFs) cultured ex vivo at defined population doublings (PDs). Early-passage fibroblasts (PD26) were used to represent proliferative cells, whereas late-passage fibroblasts (PD64) were used to represent senescent cells.

Only HFF samples were included to avoid confounding effects related to fibroblast strain heterogeneity. Three biological replicates were available for each condition (PD26 and PD64), and all downstream analyses were conducted using these samples unless otherwise specified.

The GSE63577 dataset includes multiple fibroblast strains and experimental conditions, which introduce variability in baseline transcriptional profiles and senescence dynamics. To minimize confounding effects and ensure biological consistency, the present analysis was therefore restricted to HFF samples representing clearly defined early (PD26) and senescent (PD64) stages. This selection provides a controlled and comparable framework for identifying senescence-associated transcriptional patterns while reducing cell-type-specific variability.

### 2.2. Gene Expression Preprocessing

Gene expression values provided by the dataset were reported as RPKM (Reads Per Kilobase of transcript per Million mapped reads). Prior to analysis, expression values were log2-transformed using log2(RPKM + 1) to stabilize variance and reduce the influence of extreme values. No additional normalization was applied, as samples originated from the same experimental platform and processing pipeline.

Given that the dataset provides uniformly processed RPKM values and a limited number of biological replicates per condition, a simplified preprocessing strategy was adopted to maintain consistency across downstream analyses while minimizing the introduction of additional model assumptions. Gene identifiers were mapped using the provided gene symbol annotations. Genes with missing values across all samples were excluded from downstream analyses.

### 2.3. Differential Expression Analysis

Differential gene expression analysis was performed to identify transcriptional changes associated with replicative senescence by comparing senescent (PD64) and early-passage (PD26) fibroblasts. For each gene, differences in log2-transformed expression values were assessed using Welch’s two-sample *t*-test, which does not assume equal variances between groups.

This approach was selected to provide a transparent and interpretable comparison of expression differences under small-sample conditions (*n* = 3 per group), where more complex count-based models may be sensitive to parameter estimation and underlying distributional assumptions.

Log2 fold change (log2FC) values were calculated as the difference between the mean expression in senescent and early-passage samples. *p*-values were adjusted for multiple testing using the Benjamini–Hochberg false discovery rate (FDR) procedure. Genes with positive log2FC values were considered upregulated in senescent cells. The complete differential expression results, including log2FC, *p*-values, FDR-adjusted *p*-values, and mean expression values for each condition, are provided in Supplementary Table S1.

### 2.4. Selection of Senescence-Associated Secretory Phenotype (SASP) Genes

To investigate coordinated inflammatory signaling associated with senescence, a literature-curated panel of senescence-associated secretory phenotype (SASP) genes was assembled. This panel included cytokines, chemokines, NF-κB-responsive genes, extracellular matrix-remodeling enzymes, and stress-associated factors that have been previously reported to contribute to the SASP. The curated SASP gene list and functional categorization are provided in Supplementary Table S2. Only genes present in the GSE63577 HFF expression matrix were included in downstream visualization and analysis.

### 2.5. Visualization of SASP Gene Expression

Targeted comparisons of selected SASP genes were visualized using expression plots comparing PD26 and PD64 samples. For broader assessment of SASP coordination, expression values of curated SASP genes were Z-score-normalized on a per-gene basis and visualized using heatmaps. Z-score normalization was applied to emphasize relative expression differences across samples rather than absolute expression levels.

### 2.6. Correlation and Network Analysis

To assess transcriptional associations between IL-1 signaling components, NF-κB-related genes, and SASP factors, Pearson correlation analysis was performed using log2-transformed expression values. Correlations were calculated between IL1α and IL1β, selected NF-κB pathway genes, and curated SASP genes across fibroblast samples.

Correlation coefficients (r) and corresponding *p*-values were computed for all tested gene pairs. *p*-values were adjusted for multiple testing using the Benjamini–Hochberg procedure where applicable. Correlations with an absolute correlation coefficient |r| ≥ 0.6 and *p* < 0.05 were considered biologically relevant and were used to construct correlation-based networks.

Given the limited number of samples and the comparison across distinct biological states, correlation-based networks are interpreted as reflecting transcriptional co-variation patterns rather than direct regulatory relationships.

Network visualization was performed to highlight hub genes and transcriptional connectivity. In these networks, *IL1A* and *IL1B* were treated as central nodes, NF-κB pathway components as intermediate nodes, and SASP effector genes as peripheral nodes. Full correlation statistics and network inclusion criteria are reported in Supplementary Table S3.

### 2.7. Curated Pathway Enrichment Analysis

To evaluate pathway-level organization of senescence-associated transcriptional changes, a hypothesis-driven curated pathway enrichment analysis was conducted. Pathway gene sets representing IL-1 signaling, NF-κB signaling, cytokine signaling, and inflammatory response pathways were assembled based on established pathway annotations and literature reports.

Genes upregulated in senescent fibroblasts were tested for over-representation within each curated pathway using Fisher’s exact test, with the set of expressed genes in the dataset serving as the background universe. Enrichment significance was evaluated using nominal *p*-values, and results were visualized as −log10(*p*-value) bar plots.

Upregulated genes were defined based on positive log2 fold change and nominal statistical significance (*p* < 0.05), without applying an additional fold-change cutoff. This enrichment analysis was designed as a hypothesis-driven, confirmatory approach to support interpretation of predefined inflammatory pathways rather than to perform unbiased pathway discovery. The curated pathway gene sets and enrichment results are provided in Supplementary Tables S4 and S5, respectively.

### 2.8. Statistical Analysis and Software

All statistical analyses and data processing were performed using Python (version 3.9) with standard scientific computing libraries, including pandas (v1.5.3), NumPy (v1.24.3), SciPy (v1.10.1), and statsmodels (v0.14.0). Data visualization was performed using Matplotlib (v3.7.1). Network analyses were conducted using NetworkX (v3.1). Statistical significance thresholds are specified within each analysis subsection.

### 2.9. Code and Data Availability

**Figure 1:**
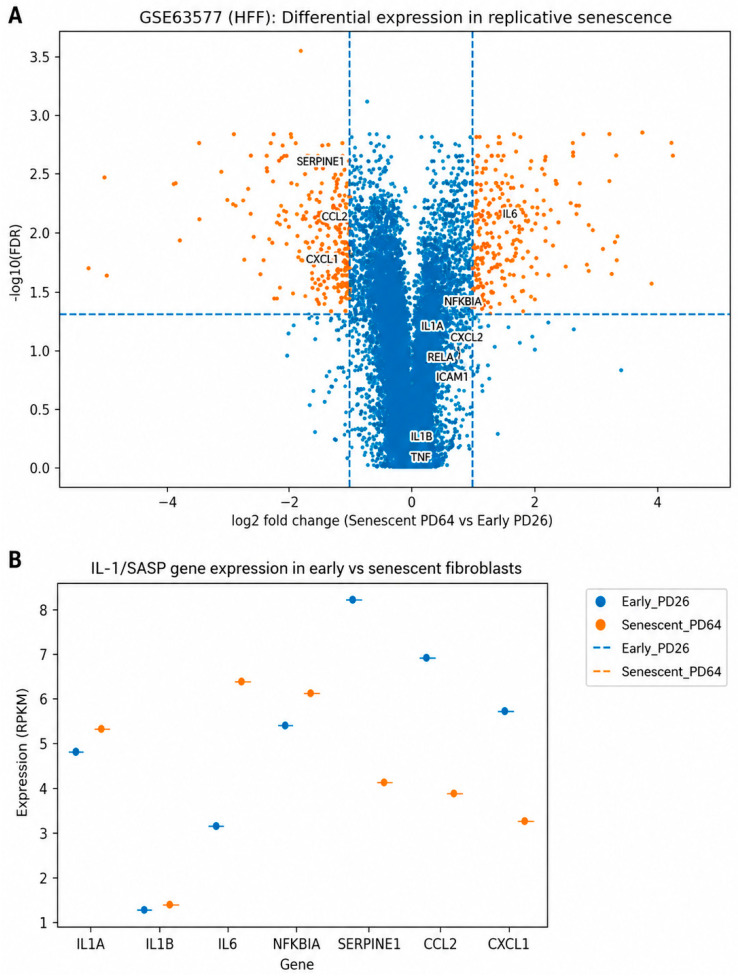
Replicative senescence is associated with an inflammatory and IL-1-associated transcriptional program in human fibroblasts. (**A**) Volcano plot depicting differential gene expression between senescent (PD64) and early-passage (PD26) human foreskin fibroblasts (HFFs) from the GSE63577 dataset. Each point represents a single gene, plotted as log2 fold change (senescent vs. early) versus −log10 false discovery rate (FDR). Genes meeting the significance threshold (FDR < 0.05) are highlighted. Selected inflammation- and SASP-associated genes, including IL-6, IL-1A, IL-1B, NFKBIA, SERPINE1, CCL2, and CXCL1, are labeled to illustrate senescence-associated transcriptional shifts. (**B**) Targeted comparison of normalized expression levels (RPKM) for key IL-1 signaling and SASP-related genes in early-passage (PD26) and senescent (PD64) fibroblasts. Data show increased expression of inflammatory mediators and downstream NF-κB-associated genes in senescent cells, consistent with activation of a senescence-associated secretory phenotype. Together, these analyses suggest that replicative senescence is associated with coordinated upregulation of inflammatory and IL-1-linked transcriptional programs in human fibroblasts.

**Figure 2:**
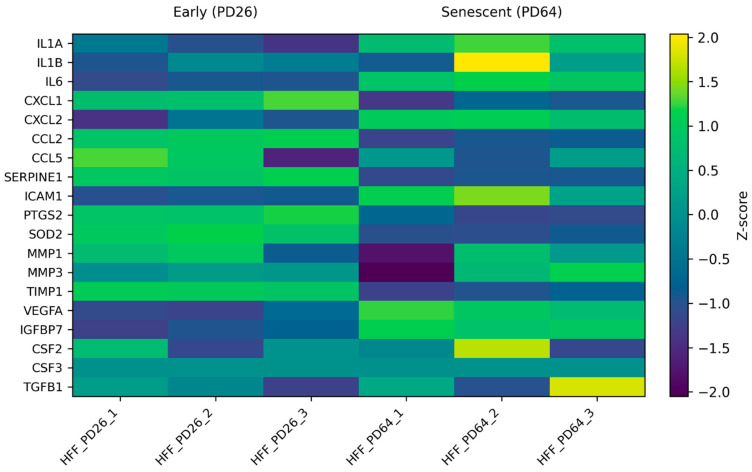
Coordinated expression patterns of senescence-associated secretory phenotype (SASP) genes during replicative senescence. Heatmap illustrating the expression patterns of a literature-curated SASP gene set in early-passage (PD26) and senescent (PD64) human foreskin fibroblasts (HFFs) from the GSE63577 dataset. Expression values were log2-transformed (RPKM + 1) and Z-score-normalized per gene to highlight relative changes across samples. Genes are displayed as rows and individual samples as columns. The heatmap shows a coordinated pattern of increased expression of pro-inflammatory cytokines, chemokines, matrix-remodeling enzymes, and regulatory factors in senescent fibroblasts, consistent with activation of a senescence-associated secretory phenotype. Group labels indicate early and senescent conditions, highlighting global transcriptional differences associated with replicative senescence.

**Figure 3:**
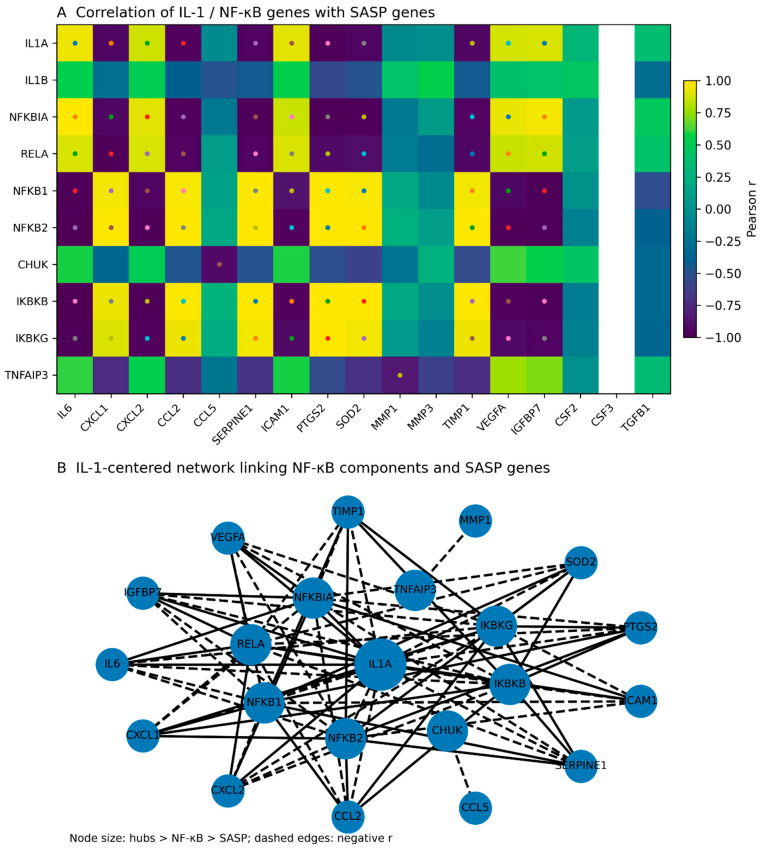
IL-1 signaling components are strongly associated with the SASP transcriptional network. (**A**) Heatmap showing Pearson correlation coefficients between IL-1 signaling and NF-κB-related genes (rows) and a literature-curated SASP gene set (columns) across human foreskin fibroblast samples from the GSE63577 dataset. Correlations were calculated using log2-transformed expression values (RPKM + 1) from early-passage (PD26) and senescent (PD64) cells combined to capture shared transcriptional associations. Color intensity represents the strength and direction of correlation (r), with positive and negative associations indicated by the color scale. Dots mark correlations that reached statistical significance (*p* < 0.05). (**B**) Correlation-based network representation showing the central, hub-like positioning of IL-1A and IL-1B within the SASP-associated transcriptional landscape. Nodes represent genes, with IL-1 cytokines shown as central hub-like nodes, NF-κB pathway components forming an intermediate layer, and SASP effector genes distributed peripherally. Edges denote significant correlations (|r| ≥ 0.6, *p* < 0.05), with solid lines indicating positive correlations and dashed lines indicating negative correlations. Node size reflects functional category (IL-1 hubs > NF-κB components > SASP genes). Together, these analyses suggest that IL-1 signaling is associated with a central, hub-like position within the SASP transcriptional network and is consistent with a layered association between IL-1 signaling, NF-κB-related genes, and SASP gene expression during replicative senescence.

**Figure 4:**
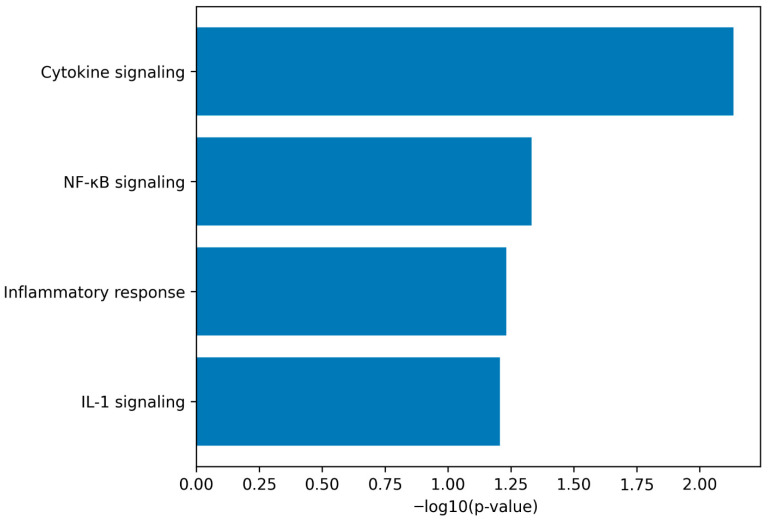
Inflammatory and NF-κB-related pathways are prominently represented in the senescence transcriptome. Bar plot summarizing hypothesis-driven, curated pathway enrichment analysis performed on genes upregulated in senescent (PD64) compared with early-passage (PD26) human foreskin fibroblasts (HFFs) from the GSE63577 dataset. Gene expression values were log2-transformed (RPKM + 1), and upregulated genes were defined based on positive log2 fold change with nominal statistical significance. Over-representation analysis was conducted using Fisher’s exact test against a curated background of expressed genes. The *x*-axis indicates −log10(*p*-value) for each pathway. Pathways associated with IL-1 signaling, NF-κB signaling, cytokine signaling, and inflammatory response show significant enrichment, supporting a pathway-level pattern consistent with IL-1-associated and inflammatory transcriptional activity in senescent fibroblasts.

All scripts, analysis notebooks, and intermediate files used to generate the results and figures in this study are provided as a single compressed supplementary file. This package includes the original expression matrix obtained from GEO (GSE63577), curated gene lists, Python scripts, and Jupyter notebooks (v6.1) corresponding to Figure 1, Figure 2, Figure 3 and Figure 4, as well as the supplementary tables. All analyses were performed using publicly available data and standard Python-based scientific computing libraries.

## 3. Results

### 3.1. Replicative Senescence Is Associated with Widespread Inflammatory Transcriptional Remodeling

To characterize transcriptional changes associated with replicative senescence, we compared the gene expression profiles of early-passage (PD26) and senescent (PD64) human foreskin fibroblasts (HFFs) from the GSE63577 dataset. Differential expression analysis revealed extensive transcriptional remodeling accompanying senescence, with numerous genes exhibiting significant upregulation in senescent cells (Figure 1A; Supplementary Table S1).

Notably, several inflammation- and senescence-associated genes were among the most prominently altered transcripts. These included cytokines and chemokines such as IL-6, CCL2, and CXCL1, as well as regulatory and stress-associated factors including SERPINE1 and NFKBIA. Components of the IL-1 signaling axis (IL-1A and IL-1B) also demonstrated increased expression or upward trends in senescent cells, consistent with prior observations linking IL-1 signaling to senescence-associated inflammatory states. Together, these findings suggest that replicative senescence is associated with robust activation of inflammatory gene expression programs.

### 3.2. Senescent Fibroblasts Display Coordinated Activation of a Senescence-Associated Secretory Phenotype

To determine whether senescence-associated transcriptional changes reflect a coordinated secretory program rather than isolated gene-level effects, we next examined expression patterns of a literature-curated senescence-associated secretory phenotype (SASP) gene set (Supplementary Table S2). Visualization of these genes revealed clear and consistent differences between early-passage and senescent fibroblasts.

A focused comparison of representative SASP-related genes demonstrated increased expression of inflammatory cytokines (IL-6), IL-1 pathway components (IL-1A, IL-1B), NF-κB-responsive genes (NFKBIA), and canonical SASP markers involved in extracellular matrix remodeling and chemokine signaling (SERPINE1, CCL2, CXCL1) in senescent cells (Figure 1B).

Expanding this analysis to the full curated SASP panel, heatmap visualization revealed a coordinated pattern of increased expression of cytokines, chemokines, matrix-remodeling enzymes, and stress-associated regulators in senescent fibroblasts relative to early-passage cells (Figure 2). Z-score normalization highlighted consistent expression shifts across senescent samples, supporting the presence of an organized SASP-associated transcriptional pattern rather than stochastic gene activation. These results suggest that replicative senescence in human fibroblasts is characterized by concerted activation of SASP genes.

### 3.3. IL-1 Signaling Components Are Strongly Associated with the SASP Transcriptional Network

Given the prominent involvement of IL-1-related genes in senescence-associated transcriptional changes, we next examined whether IL-1 signaling is positioned in a central, hub-like manner within the SASP-associated transcriptional network. Pearson correlation analysis was performed to assess transcriptional associations between IL-1 cytokines (IL-1A and IL-1B), NF-κB-related signaling components, and SASP genes across fibroblast samples.

Correlation heatmap analysis revealed widespread and significant associations between IL-1/NF-κB pathway genes and SASP factors (Figure 3A; Supplementary Table S3). Both IL-1A and IL-1B exhibited strong positive correlations with multiple inflammatory cytokines, chemokines, and matrix-remodeling genes, indicating tight transcriptional coupling. NF-κB-related genes, including NFKBIA, RELA, and TNFAIP3, also showed extensive correlations with SASP components, consistent with known roles of NF-κB in senescence-associated inflammatory signaling.

Network-based visualization further showed that IL-1A and IL-1B occupy central hub-like positions, linking NF-κB signaling components to downstream SASP effector genes (Figure 3B). The network structure is consistent with a layered organization in which IL-1 cytokines connect to NF-κB pathway genes, which in turn are associated with a broad range of SASP factors. Collectively, these analyses suggest that IL-1 signaling is associated with a central, hub-like position within the SASP-associated transcriptional network during replicative senescence.

### 3.4. Inflammatory and NF-κB-Related Pathways Dominate the Senescence Transcriptome

To determine whether gene- and network-level findings were reflected at the pathway level, we performed a hypothesis-driven curated pathway enrichment analysis focusing on inflammatory signaling cascades. Genes upregulated in senescent fibroblasts were tested for over-representation within curated gene sets representing IL-1 signaling, NF-κB signaling, cytokine signaling, and inflammatory response pathways (Supplementary Tables S4 and S5).

This analysis revealed significant enrichment of all four inflammatory pathway categories among genes upregulated in senescent cells (Figure 4). Pathways related to IL-1 signaling and NF-κB signaling showed particularly strong enrichment, consistent with the transcriptomic associations observed in the network analysis (Figure 3). Cytokine signaling and inflammatory response pathways were likewise prominently represented, supporting the interpretation that replicative senescence is associated with coordinated activation of inflammatory transcriptional programs.

Together, these pathway-level results integrate gene-level, network-level, and systems-level analyses, suggesting that the senescence transcriptome in human fibroblasts is characterized by prominent IL-1-associated and NF-κB-related inflammatory signaling patterns. Given the hypothesis-driven and curated nature of this analysis, these findings should be interpreted as confirmatory and supportive of the observed transcriptional associations rather than as evidence of independent pathway discovery.

### 3.5. Validation of IL-1/SASP Transcriptional Associations in an Independent Dataset

To assess the robustness and generalizability of the IL-1/SASP-associated transcriptional patterns identified in GSE63577, we analyzed an independent transcriptomic dataset of human fibroblast replicative senescence (GSE41714). This dataset comprises a time-resolved series of fibroblast samples characterized by increasing population doubling times, allowing comparison between proliferative and advanced senescence-stage cells.

For validation, we compared early proliferative fibroblasts (doubling time 2–3 days) with advanced senescence-stage fibroblasts (doubling time 14–20 days). Expression levels of key IL-1 signaling components (*IL1A* and *IL1B*) and representative SASP-associated genes (IL6, CCL2, and CXCL1) were examined.

**Figure 5:**
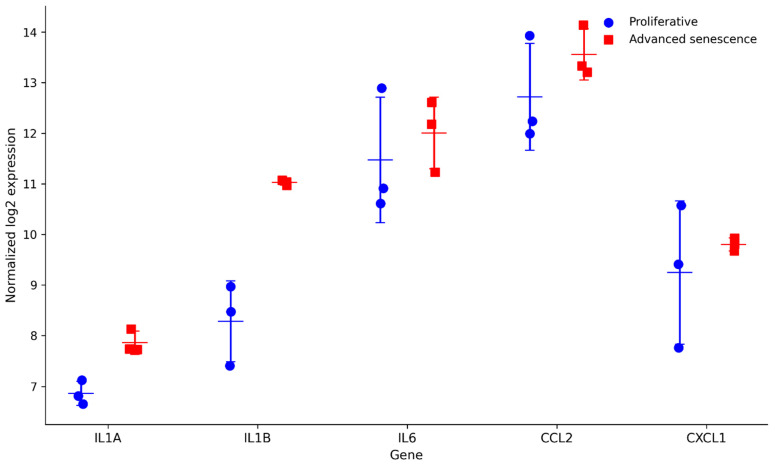
Validation of IL-1/SASP-associated transcriptional patterns in an independent fibroblast senescence dataset. Normalized log2 expression values of *IL1A, IL1B*, and representative senescence-associated secretory phenotype (SASP) genes (IL6, CCL2, and CXCL1) were examined in an independent human fibroblast replicative senescence dataset (GSE41714). Proliferative fibroblasts (doubling time 2–3 days) were compared with advanced senescence-stage fibroblasts (doubling time 14–20 days). Each point represents an individual biological sample, and horizontal bars indicate mean ± standard deviation. Consistent with the primary dataset (GSE63577), IL-1 signaling components and selected SASP-associated genes exhibit higher expression in advanced senescence-stage fibroblasts, supporting the consistency of the observed transcriptomic associations across datasets.

Consistent with observations from the primary dataset, *IL1A* and *IL1B* expression levels were higher in advanced senescence-stage fibroblasts compared with proliferative cells (Figure 5). Similarly, SASP-associated genes, including IL6, CCL2, and CXCL1, exhibited increased expression in senescent samples, although the magnitude of change varied across genes.

Importantly, these findings are consistent with the overall directionality of the IL-1-associated and SASP-related transcriptional changes observed in GSE63577, supporting the reproducibility of the identified transcriptomic associations across independent experimental systems. While these results do not establish causal regulatory relationships, they provide additional evidence that IL-1 signaling components are consistently associated with the broader SASP transcriptional program in human fibroblast senescence.

## 4. Discussion

In this focused in silico study, we interrogated a well-established human fibroblast replicative senescence transcriptome (GSE63577) to test whether IL-1 signaling is associated with and positioned within a curated SASP transcriptional program. Collectively, our results converge on a coherent model in which senescent fibroblasts show broad inflammatory remodeling with strong induction of canonical SASP factors, while IL-1 pathway components and NF-κB-associated genes form a highly connected module prominent in pathway-level interpretations of the senescence transcriptome. These findings are consistent with the modern view of the SASP as a heterogeneous but mechanistically structured program, where a subset of inflammatory regulators repeatedly acts as “control nodes” shaping downstream cytokine and chemokine expression [7,19,20].

A principal observation from our differential expression analysis (Figure 1A) is that replicative senescence in primary human fibroblasts is accompanied by a broad, coordinated shift toward inflammatory and secretory gene expression. This aligns with the established concept that senescence is not merely a growth arrest but a profound phenotypic state characterized by a hypersecretory program that drives local and systemic effects [19]. Notably, Figure 1B and the curated SASP heatmap (Figure 2) demonstrate that a literature-defined set of SASP-associated genes exhibits clear, directionally consistent expression changes between early and late population doublings, providing transcript-level evidence that classical SASP components are robustly embedded within the fibroblast senescence state in this dataset. This observation complements large-scale profiling efforts showing that while the SASP is context-dependent, a reproducible “core” of inflammatory mediators tends to recur across senescence contexts and is often detectable at the transcriptomic and proteomic levels [21].

A key contribution of this work is the explicit network-oriented framing (Figure 3), which supports the interpretation that IL-1 signaling components are not merely upregulated alongside other inflammatory transcripts, but are associated with a tightly connected correlation structure involving canonical SASP and NF-κB-related genes. This transcriptomic connectivity is mechanistically plausible: IL-1 signaling has long been implicated as an upstream amplifier of the senescence-associated cytokine network. In classic experiments, cell surface-associated IL-1A was shown to be essential for the senescence-associated IL-6/IL-8 network in human fibroblasts, placing IL-1A upstream of core SASP cytokines [8,22]. In parallel, NF-κB has been described as a master transcriptional regulator of SASP output, influencing a substantial fraction of SASP-associated gene expression in fibroblast senescence models [8,23]. Furthermore, inflammasome-mediated IL-1 signaling has been demonstrated to control key aspects of paracrine senescence and SASP induction, reinforcing IL-1 pathway activity as a central regulatory lever rather than a passive output [24]. Our correlation/network results are consistent with these mechanistic frameworks and provide dataset-specific transcriptomic evidence that IL-1A/IL-1B and NF-κB-associated transcripts form an integrated module linked to the SASP gene program.

Importantly, these findings should be interpreted within the constraints of correlation analyses: co-expression does not establish causal direction. Nonetheless, the convergence of (i) robust SASP induction (Figure 1 and Figure 2), (ii) strong association of IL-1 pathway components with SASP transcripts (Figure 3), and (iii) pathway-level enrichment emphasizing inflammatory and cytokine signaling (Figure 4) provides a cohesive, biologically consistent narrative in which IL-1/NF-κB circuitry is consistently embedded within the senescence-associated transcriptional landscape.

In addition, analysis of an independent fibroblast senescence dataset (GSE41714) demonstrated consistent IL-1-associated and SASP-related transcriptional trends, supporting the reproducibility of these transcriptomic associations across experimental systems.

Our curated pathway integration (Figure 4) reinforces the interpretation that inflammatory and cytokine-driven processes are prominently represented in the senescence transcriptome in this fibroblast model. This conclusion fits well with contemporary reviews emphasizing that SASP programs are shaped by stress-response signaling (including DNA damage responses) and transcriptional regulators such as NF-κB and C/EBP family factors, yielding sustained expression of inflammatory mediators that can become pathological when senescence is chronic [19]. It also aligns with broader perspectives on senescence as a therapeutically targetable state, either by eliminating senescent cells (senolytics) or by suppressing harmful SASP components (senomorphics/SASP inhibitors) [11,20,25,26]. Of note, multiple mechanistic layers implicated in SASP control intersect with IL-1 signaling, including translational regulation: mTOR has been shown to regulate pro-tumorigenic SASP output by promoting IL-1A translation, and mTOR inhibition can attenuate inflammatory SASP features in senescent cells [27]. This provides a plausible mechanistic bridge between pathway enrichment emphasizing inflammatory signaling and the emerging therapeutic literature that positions mTOR and NF-κB/IL-1-related axes as actionable SASP regulators [11,20,25,26,27,28].

A practical implication of identifying IL-1/NF-κB-linked transcriptional modules in senescent fibroblasts is that these axes intersect with disease-relevant inflammatory biology beyond senescence itself. Persistent IL-1β signaling has been implicated in shaping tissue microenvironments in chronic inflammatory diseases and cancer, with consequences for immune recruitment and remodeling [29,30]. While the present study does not evaluate tumor or tissue outcomes, our network-level and pathway-level findings support the rationale that IL-1-centered inflammatory programs within senescent fibroblasts may contribute to broader inflammatory milieus relevant to aging and disease progression. In this sense, the transcriptomic embedding of IL-1 signaling within the SASP network provides a conceptual framework for linking senescence biology to chronic inflammation phenotypes discussed in the wider literature [19,28,29,30,31,32,33].

## 5. Limitations and Future Directions

Several limitations should be noted for the present study. First, this is an in silico analysis of transcriptomic data; mRNA changes do not always translate into protein abundance or secreted factor levels, and the SASP is ultimately a secretome. Integrating transcript-level findings with proteomic SASP resources (e.g., SASP Atlas) may sharpen biological interpretation, particularly for factors with strong post-transcriptional regulation [21]. Second, correlation-based network structure can reveal coordinated programs but cannot infer causality or directionality. Additionally, the relatively small sample size of the primary dataset (*n* = 3 per condition) may limit statistical power and the stability of correlation estimates, and restricts the extent to which the findings can be broadly generalized. Furthermore, the present analysis focuses on a single fibroblast strain (human foreskin fibroblasts, HFFs), and therefore does not capture potential variability across different fibroblast types, tissues, or senescence-inducing conditions, which may influence SASP composition and regulatory architecture. Third, our pathway integration was curated rather than fully hypothesis-free; while this improves interpretability and aligns with the study aim, it may underrepresent additional pathways relevant to senescence biology in this dataset.

An additional limitation relates to the selection of population doubling (PD) stages used for comparison. While PD26 and PD64 were chosen to represent early and senescent states within a controlled and internally consistent framework, alternative comparisons available within the dataset (e.g., PD16 versus PD74) may provide a more sharply defined proliferative versus advanced senescence contrast. The present study therefore prioritizes a reductionist and controlled comparison strategy aimed at minimizing confounding variability, but this choice may limit the extent to which the observed transcriptional patterns fully capture the broader dynamic range of replicative senescence. Consequently, the findings should be interpreted as system-specific transcriptomic associations that warrant further validation across additional PD stages and experimental contexts.

Future work can extend these results in three practical directions. (i) External validation: repeating the same IL-1/SASP network and pathway analysis across independent fibroblast senescence datasets would test generalizability and robustness. In particular, extending analyses to additional fibroblast strains, alternative PD comparisons, and diverse senescence models would help determine the extent to which the observed IL-1/SASP network structure represents a conserved versus context-dependent feature. (ii) Perturbation-aware inference: integrating datasets where IL-1 signaling is experimentally modulated (e.g., IL-1R blockade, NF-κB inhibition, or mTOR inhibition) would more directly connect the observed transcriptional associations with causal control of SASP programs [25,27,34]. (iii) Translational framing: from a senomorphic perspective, targeted suppression of SASP regulators, potentially including IL-1 pathway targeting strategies that already exist clinically (e.g., IL-1 receptor antagonism), may be conceptually aligned with efforts to reduce chronic SASP-driven inflammation without removing senescent cells [25,35]. At minimum, these avenues highlight how identifying hub-like inflammatory modules at the transcriptomic level can motivate mechanistically grounded intervention hypotheses.

## 6. Conclusions

Collectively, the analysis performed in this study supports a unified interpretation that replicative senescence in human fibroblasts is characterized by robust SASP gene induction and a tightly connected IL-1/NF-κB-associated transcriptional module at the level of transcriptomic association. These results add transcriptomic network-level evidence to prior mechanistic models placing IL-1 signaling upstream of key SASP outputs and reinforce pathway-level interpretations in which inflammatory and cytokine signaling are prominently represented in the senescent fibroblast state [22,23,24,27]. By presenting a compact, reproducible, single-dataset analysis supported by validation in an independent dataset, this study contributes a clear systems-level narrative relevant to both basic senescence biology and the growing interest in strategies targeting harmful SASP outputs [19,20,25,26]. 

## Data Availability

The RNA-sequencing dataset analyzed in this study is publicly available in GEO under accession number GSE63577. All scripts, analysis notebooks, curated gene lists, and supplementary tables used for data preprocessing, differential expression analysis, network analysis, pathway enrichment, and figure generation are provided as a Supplementary Materials accompanying this article. This package enables full reproduction of the analyses and figures presented in the manuscript.

## Supplementary Materials

The following supporting information can be downloaded at: https://www.mdpi.com/article/10.3390/genes17050527/s1. Table S1: Differential expression results for senescent (PD64) versus early-passage (PD26) fibroblasts. Table S2: Curated senescence-associated secretory phenotype (SASP) gene list. Table S3: Correlation analysis results for IL-1, NF-κB, and SASP genes. Table S4: Curated pathway gene sets used for enrichment analysis. Table S5: Pathway enrichment analysis results. Supplementary File S1: Supplementary_Code_GSE63577_IL1_SASP_Analysis.
